# Towards Advanced Materials: Functional Perspectives of Co-Doped ZnO Thin Films

**DOI:** 10.3390/mi16101179

**Published:** 2025-10-18

**Authors:** Mariuca Gartner, Mariana Chelu, Anna Szekeres, Peter Petrik

**Affiliations:** 1 Institute of Physical Chemistry “Ilie Murgulescu”, 202 Splaiul Independentei, 060021 Bucharest, Romania; mgartner@icf.ro; 2 Institute of Solid State Physics, Bulgarian Academy of Sciences, 72 Tsarigradsko Chaussee, 1784 Sofia, Bulgaria; szekeres@issp.bas.bg; 3 Hungarian Research Network, Centre for Energy Research, Konkoly Thege Street 29–33, 1121 Budapest, Hungary; petrik.peter@ek.hun-ren.hu; 4 Department of Electrical Engineering, University of Debrecen, Bem Square 18, 4026 Debrecen, Hungary

**Keywords:** ZnO thin films, co-doping, gas sensors, biosensors, optical applications, solar energy harvesting

## Abstract

Zinc oxide (ZnO) thin films have attracted increasing attention as promising materials for sensing applications due to their wide band gap, high exciton binding energy, and remarkable chemical stability. However, the inherent limitations of pure ZnO, such as moderate sensitivity, selectivity, and relatively high operating temperatures, limit its widespread use in advanced sensing technologies. Co-doping, or dual doping with two distinct elements, has emerged as an effective strategy to overcome these challenges by synergistically tailoring the structural, electronic, and surface properties of ZnO thin films. This review provides a comprehensive overview of recent advances in the development of co-doped ZnO thin films for sensing applications. The focus is on the role of different combinations of dopants, including transition metals, rare earth elements, and non-metals, in modulating the charge carrier concentration, oxygen vacancy density, and adsorption dynamics. These effects collectively enhance the sensing properties and long-term stability and reduce detection limits. The analysis highlights the correlations between synthesis methods, dopant incorporation mechanisms, and resulting sensor performance. Key challenges such as dopant clustering, reproducibility, and scalability are discussed, along with emerging opportunities in flexible room-temperature sensor platforms. Overall, it has been demonstrated that co-doped ZnO thin films represent a versatile and tunable class of sensing materials with strong potential for next-generation environmental and biomedical monitoring.

## 1. Introduction

With the advent of industrial civilization, human development has grown heavily reliant on fossil fuels and material resources. This dependence has caused extensive ecological damage and environmental crises, contributing to causing global climate change. Current global warming, primarily driven by massive emissions of greenhouse gases, especially carbon dioxide, has profoundly affected both the environment and human life. The proposal to achieve carbon neutrality before 2050, discussed at the 75th General Debate of the United Nations General Assembly, together with other measures, could significantly contribute to reducing carbon emissions and mitigating global climate [1,2].

In this context, the scientific community has undertaken extensive efforts across diverse disciplines to mitigate natural disasters and to address the escalating crises of climate change and biodiversity loss. These endeavors are reflected in a substantial body of published research focused on the development of advanced materials, innovative techniques, and emerging nanotechnologies [3,4,5].

The continuous advancement of functional materials is crucial for developing high-performance sensors, energy harvesting devices, and optical technologies [6,7,8]. Thin-film semiconductors play a central role in these fields, providing scalable, tunable, versatile platforms for integrated devices [9,10,11,12,13,14,15].

Zinc oxide (ZnO) is a wide-bandgap semiconductor (∼3.37 eV at room temperature) with a high exciton binding energy (∼60 meV), excellent chemical stability, high optical transparency, and excellent piezoelectric properties [16,17,18,19]. These characteristics make it very attractive for applications in optoelectronics, gas sensing, transparent electronics, and UV photodetectors [20,21,22,23,24,25,26]. In particular, ZnO thin films have been extensively studied as sensitive materials for chemical and biological sensors due to their high surface-to-volume ratio, good thermal stability, and ease of fabrication [27,28,29,30,31,32,33,34].

However, the sensing performance of pure ZnO, such as response magnitude, selectivity, and response/recovery speed, is often limited by its relatively low intrinsic conductivity, limited number of active sites, and high operating temperatures [35,36,37,38]. In addition, pristine ZnO thin films exhibit several limitations, including (i) incomplete control over electrical conductivity and carrier type; (ii) high density of intrinsic defects that act as non-radiative recombination centers; and (iii) limited tunability of optical and surface properties for application-specific requirements [39,40,41].

Extensive research has demonstrated that doping represents one of the most effective strategies for enhancing charge carrier concentration and thereby improving the functional performance of ZnO materials [42,43,44]. The beneficial influence of doping can be primarily attributed to three decisive effects: (a) the narrowing of the band gap, which facilitates enhanced light absorption and surface adsorption; (b) the improvement of electrical conductivity and carrier mobility within the ZnO matrix; and (c) the modulation of the relative positions of the conduction band (CB) and valence band (VB), which alters the electronic structure and optimizes charge transfer pathways.

To address these limitations and challenges, researchers have explored a range of targeted doping strategies, in which extrinsic elements are deliberately incorporated into the ZnO lattice to modify its structural, electronic, and optical characteristics [45,46,47,48,49]. More recently, co-doping (or dual doping) of ZnO thin films with two different elements has emerged as a powerful approach to achieve synergistic improvements in material performance for various applications, especially in sensing, solar energy harvesting, and optical technologies [50,51,52,53,54,55,56,57]. The reliability of ZnO materials modified through single-element doping has been shown to be limited, due to issues such as dopant instability, compensation effects, and uncontrollable defect states, thereby motivating the advancement of co-doping strategies [50].

In this review, we explore recent progress in the field of electronic materials integrated into semiconductors, focusing on the fabrication, characterization, and properties of nanocrystalline ZnO thin films, as well as on the architecture of electronic devices. We will briefly discuss the importance of co-doping of ZnO films and demonstrate their versatility across numerous applications such as bio- and gas-sensors, solar energy harvesting, and optoelectronics devices.

## 2. Why Co-Dope ZnO?

### 2.1. Single Versus Dual Doping

Although doping with a single element can improve certain properties, such as Al, Ga, or In increasing n-type conductivity or Co and Mn introducing magnetic or optical activity, it often leads to compromises [58,59,60,61,62]. For example, an excessive concentration of charge carriers can reduce transparency, while too many defects can degrade luminescence [63,64,65,66].

Dual doping by introducing two different elements, such as aluminum and samarium (Sm), terbium (Tb), ytterbium (Yb), or erbium (Er), offers a way to balance, with synergistic effects, and simultaneously optimize multiple properties, thus improving the material performance for specific applications [67,68].

Co-doped ZnO thin films can be used in gas sensors, UV detectors, and other types of sensors due to their tunable electrical conductivity and sensitivity to various stimuli [69]. For example, in sensing applications, doping ZnO with co-dopants can have several beneficial effects, such as the following:

Defect engineering: Co-dopant ions can replace Zn sites or occupy interstitial sites, introducing oxygen vacancies and zinc interstitials that serve as active sites for gas adsorption.

Enhanced conductivity: Dual doping can increase the concentration and mobility of charge carriers, reduce film resistance, and improve the signal-to-noise ratio in sensing.

Bandgap tuning: Slight changes in the optical bandgap can influence surface reactivity and photon-assisted sensing mechanisms.

Magnetic and catalytic effects: Co-dopant ions can exhibit catalytic activity and magnetic interactions, enhancing sensitivity to specific gases [70,71].

In solar energy harvesting applications, co-doped ZnO films can serve as transparent conductive oxides (TCOs) in solar cells, improving their efficiency and performance [72,73]. In the case of optical technologies, the ability to tune the bandgap and optical properties of ZnO through doping makes it suitable for applications in light-emitting diodes (LEDs), photodetectors, and optical coatings.

To fine-tune the electrical and optical properties of ZnO, different combinations of dopants, such as cation–cation or cation–anion doping, have been explored [74,75,76]. For instance, fluorine–aluminum-co-doped ZnO (FAZO) films have demonstrated promising results in high-efficiency solar cells due to their improved electrical and optical performance. When ZnO was co-doped with samarium and aluminum, the films exhibited high transparency and low electrical resistivity, making them suitable for optoelectronic devices. Other design strategies have focused on co-doping ZnO with terbium and erbium. This approach can modify the band gap and refractive index of ZnO, potentially influencing its application in optoelectronic devices and even in anticancer treatments.

### 2.2. Enhancement Mechanisms

- Substitutional dopants modify the band structure and charge carrier concentration.
- Co-dopants passivate harmful defects and create desired luminescent centers.
- Ionic radius and valence mismatch of dopants strain the lattice in a controlled manner, improving crystallinity or introducing active sites.

### 2.3. Thin Film Preparation

Several techniques have been developed to obtain ZnO thin films with suitable dopants for specific applications [22,31,50,63,66]. The sol–gel spin/dipping deposition is a commonly applied eco-friendly method for producing cost-effective coatings over large areas [51,59,69]. Doped nanostructured coatings can also be prepared using environmentally friendly precursors via hydrothermal synthesis at low temperatures (below 100 °C), further developed from sol–gel synthesis [31,77,78]. Pulsed laser deposition (PLD) is used to produce thin films with controllable stoichiometry and dopants concentration by fine-tuning the reactive environment [66,70,79]. The RF/DC magnetron sputtering method provides uniform, high-quality films with precise control [40,55,64,71,80]. Spray pyrolysis [20,53,54,81] and ALD [19] are typically used for scalable and conformal coatings. Chemical vapor deposition (CVD) is also widely employed in various forms, including low-pressure CVD, plasma-enhanced CVD [82], and aerosol-assisted CVD [83,84].

The optimization of dual doping in ZnO thin films requires careful regulation of dopant concentrations to avoid phase separation and suppress the formation of dopant clusters. Furthermore, post-deposition annealing is a crucial step, as it enhances the crystallinity of the films, facilitates the activation of dopant species, and effectively mitigates structural defects typically introduced during chemical vapor deposition (CVD).

**Figure 1:**
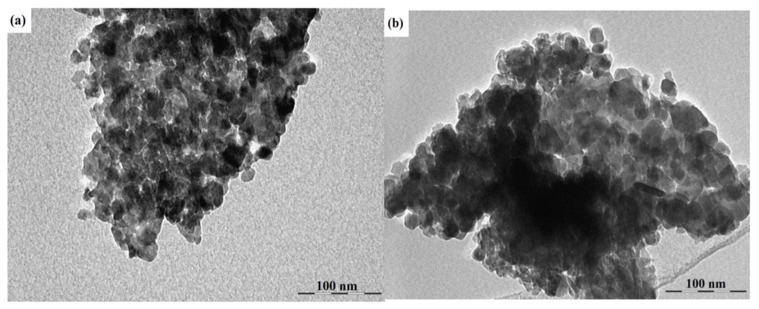
TEM images of (**a**) 5%Yb, 1%Er-doped ZnO and (**b**) 5%Yb, 3%Er-doped ZnO. Reproduced from [81].

As an example, the research in [81] shows how crucial the method of obtaining ZnO nanostructures and the level of their doping is. Using the spray pyrolysis method on glass substrates, zinc oxide in the form of thin layers doped and co-doped with rare earth ions of the Yb_x_Er_y_ZnO type were obtained, with relatively low dopant concentrations (x = 5% and y = 0, 1, 3%) [81]. The resulting polycrystalline films showed a hexagonal wurtzite structure with a preferential orientation along the (002) plane. Optical characterization in the 200–800 nm range revealed that co-doping significantly influenced the transmission values. Also, photoluminescence measurements showed a well-defined infrared emission peak around 980 nm, corresponding to the transition between the ^2^F_5/2_ (ground state) and ^2^F_7/2_ (excited state) electronic levels, suggesting efficient photon transfer between the ZnO base matrix and the Yb^3+^ dopant ion. Transmission electron microscopy (TEM) revealed the formation of agglomerations of grains in the form of hexagons and spheres inserted into hexagons. The co-doped ZnO films exhibited particle sizes ranging from 9 nm to 11 nm for ZnO: 5% Yb + 1% Er and ZnO: 5% Yb + 3% Er, respectively (Figure 1). The study in [81] highlighted that even low concentrations of Yb and Er co-doping can significantly influence the properties of ZnO thin films for improved devices and advanced technological applications.

## 3. Applications of Co-Doped ZnO Thin Films

The high surface-to-volume ratio, the defects chemistry dominated by oxygen vacancy, and the semiconducting behavior of ZnO make it an excellent platform for gas, chemical, and biological sensors. Co-doping further enhances these characteristics. The main benefits of dual doping of ZnO films include

(i) Increased charge carrier density and mobility, leading to faster response.
(ii) Higher concentration of oxygen vacancies and active sites, improving sensitivity.
(iii) Customizable surface potential and adsorption properties, enhancing selectivity.
(iv) Improved stability and repeatability of detection signals.

### 3.1. Gas Sensors

Gas sensors play a crucial role in environmental monitoring, industrial safety, automotive emission control, and healthcare [85,86,87,88,89]. ZnO is a promising gas-sensing material due to its strong thermal and chemical stability [90,91].

Several approaches have been employed to enhance the gas sensing properties of ZnO, such as tailoring its morphology, modifying surface structures, and engineering intrinsic defects. Nevertheless, the sensitivity and selectivity of single-component ZnO remain limited. Co-doping with two elements, either metal or non-metal ions, has emerged as a widely adopted strategy to significantly improve the gas sensing performance of ZnO. Doubly doped ZnO thin films (e.g., Al+Co, Ga+Cu, Cr+Mn) exhibit enhanced sensitivity to both reducing gases (H_2_, CO) and oxidizing gases (NO_2_, O_3_), even at lower operating temperatures. Co-doping can generate extrinsic defects and impurity energy levels, modify the electronic structure and conductivity, and thereby create additional active sites in ZnO, leading to enhanced performance.

A variety of preparation methods are employed to achieve controlled doping, uniform microstructures, and tailored film properties. Commonly used techniques include both chemical and physical methods [92,93,94]. The performance of co-doped ZnO films also strongly depends on the choice of substrates, which affects lattice matching, film adhesion, and device integration. Commonly used substrates for gas sensors include (i) sapphire, offering excellent lattice compatibility and thermal stability for high-quality epitaxial growth; (ii) Silicon (Si), widely used due to its compatibility with microelectronic fabrication processes; (iii) glass, a cost-effective and transparent option suitable for large-area deposition; or (iv) alumina and quartz, favored for their high-temperature stability in harsh sensing environments [95,96].

The performance of gas sensors can be quantified using several key indicators:

(a) Sensitivity—relative change in resistance upon exposure to the target gas;
(b) Response and recovery time—the speed of change and return to baseline;
(c) Selectivity—the ability to distinguish target gases from interfering species;
(d) Operating temperature—lower operating temperature increases energy efficiency.

Defect engineering with two dopants can create more oxygen vacancies and donor/acceptor states, thereby facilitating gas adsorption and optimizing charge transfer.

Catalytic effects are enhanced by metal dopants (e.g., Pd, Ag, Pt), which reduce the activation energy required for surface reactions, the barrier that must be overcome for a reaction to occur [97]. By lowering this energy barrier, these metals accelerate surface reactions, improve efficiency, and provide a faster kinetic pathway for forming new chemical bonds and substances.

Co-dopants also alter the electronic structure of ZnO, allowing control of the band gap, improving charge carrier mobility, and ultimately enhancing their performance [98]. By generating new energy levels and matching charge distributions within the material, co-doping can narrow the band gap for better light absorption, increase charge carrier mobility for improved conductivity, and suppress charge carrier recombination, leading to improved photocatalytic activity or thermoelectric properties.

Doping gas sensing materials with transition metals and other elements changes their surface chemistry, generating specific active sites that increase interaction with a target gas while reducing interactions with other gases, thereby improving the selectivity of the sensors [99]. This tailoring of material properties, such as charge transfer and redox properties, promotes strong binding with the target molecule through chemisorption, allowing for more precise detection and differentiation in complex gas mixtures, and increased selectivity [100].

Catalytically active dopants can reduce the operating temperature required for gas sensors by creating new activation pathways that reduce the need for high thermal energy [101]. These dopants facilitate gas reactions and oxygen activation, improving sensor sensitivity and response time at lower temperatures, including room temperature (RT). This results in increased energy efficiency and extended catalyst lifetime across applications such as air quality monitoring and biomedical diagnostics. Moreover, eliminating the need for a built-in heater behind the sensing element can further reduce manufacturing costs [84].

Dual-noble-metal doping enhances the catalytic dissociation of H_2_ and accelerates electron transfer, resulting in faster response/recovery times at lower operating temperatures. In the case of hydrogen sensors, doping with Al and Mg significantly contributes to good catalytic activity, directly reflected in the detection performance [102].

In the case of nitrogen dioxide (NO_2_) sensors, both the synthesis methods and the doping modify the selectivity and response of the sensitive material. The introduction of elements such as Ni and Al can enhance electrical conductivity and sensitivity, making the materials suitable for detecting hydrogen leaks in fuel cells [103,104]. By introducing Cu and Ag as dopants in ZnO films, it was observed that Cu improves CO adsorption, while Ag increases the oxygen vacancy concentration, resulting in high CO selectivity. Similarly, Fe and Sn doping increases stability and reduces cross-sensitivity to interfering gases, such as H_2_ or CH_4_, improving selectivity [105,106,107,108,109,110,111,112,113,114,115,116].

### 3.2. Biosensors

Biosensors have become an essential component of modern diagnostics due to their rapid, accurate, reliable, and cost-effective detection of various analytes. Among the materials used in sensor applications, zinc oxide stands out for its excellent antibacterial efficacy against different bacterial strains [117], non-toxicity to human cells, and, not least, its high isoelectric point, which enables efficient immobilization of biomolecules [118]. The rapid development of technological methods for synthesis of ZnO nanostructures with diverse morphologies (nanowires, nanotubes, nanorods, etc.) has greatly enhanced their functionality for various applications in medical diagnostics, dentistry, pharmaceuticals, and the food and packaging industries. Most of the methods used to produce ZnO nanostructures are discussed in Section 2.3, while additional details can be found in [119,120,121], which review various synthesis approaches and classifications based on structural dimensions.

The rapid global spread of the COVID-19 infectious disease has given strong impetus for development of inexpensive, easy-to-use, and portable biosensors. In [122], different types of biosensors used in the diagnosis of viral respiratory infections were reviewed, along with perspectives on developing biosensors for rapid diagnostics to limit the spread of this kind of virus. Research has focused on more efficient methods for the rapid detection of viral analytes. So-called point-of-care (POC) biosensor devices provide rapid on-site testing and immediate results [123], making them critically important in biomedical applications. Recently, miniaturized POC biosensors have become commercially available, improving healthcare quality, particularly in developing regions with limited access to medical facilities.

**Figure 2:**
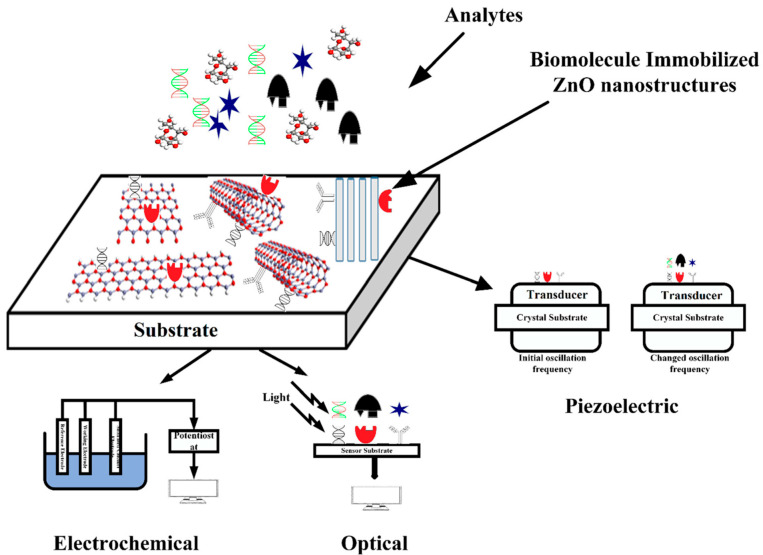
Schematic representation of the working principle of biosensors, based on ZnO nanostructures. Reprinted with permission from [123].

Over the last decade of intensive research, ZnO nanostructure-based biosensors have been developed to meet the requirements for rapid measurement, faster response time, higher stability and sensitivity, and minimal reagent use. This progress is reflected in the large number of scientific publications that have appeared, where the biosensors are classified according to the analyte type and sensing mechanism (see for example [117,118,119,120,121,122,123,124,125,126,127]). Depending on the biosensing characteristics of zinc oxide, ZnO-based devices are classified as optical, piezoelectric, and electrochemical. Figure 2, reproduced from [123], schematically illustrates the working principle of biosensors.

As a matrix layer in biosensor applications, ZnO exhibits strong binding to biomolecules, due to its high isoelectric point, and has strong sensitivity, due to its high surface-to-volume ratio, which enhance sensitivity. ZnO nanoparticles also act as excellent antibacterial agents and are effectively used as antibacterial coatings for medical devices, textiles, and food packaging materials to prevent bacterial growth and reduce the risk of infections.

In [120], the authors provided an overview on synthesis methods for ZnO nanostructures and discussed the advances in various ZnO-based biosensors for medical diagnosis, pharmaceutical analysis, food safety, and environmental pollution monitoring.

**Figure 3:**
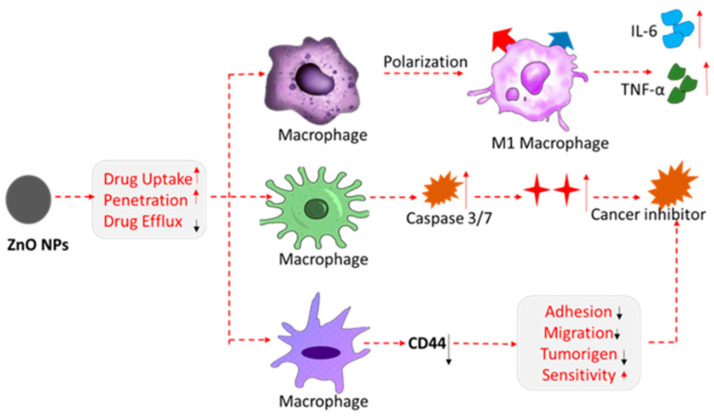
Schematic illustration of the multifunctional roles of zinc oxide nanoparticles in cancer therapy. (Arrow color denoted: red—increasing; black—decreasing). Reproduced from [128].

Similarly, [128] reviewed research from the past 5 years focusing on the multifunctional role of the biosensors in healthcare, environmental protection, and food processing industries. The recent applications of zinc oxide nanoparticles in antimicrobial (antibacterial, antifungal, antiviral) activities are summarized and supported by relevant citations in Table 1 of [128]. It has been shown that ZnO nanoparticles are successfully used in cancer treatment due to their ability to generate reactive oxygen species and to have phototoxicity under UV light. The role of the ZnO nanoparticles in cancer therapy is demonstrated in Figure 3.

From the above studies, biological sensors use mainly pure ZnO nanoparticles with different morphologies However, metal oxide-modified ZnO nanostructures have been proposed as an efficient strategy to enhance biosensor sensitivity [129].

While co-doped ZnO is widely used in gas-sensing devices, reports on its use in biological sensors are relatively limited. Dual dopants in ZnO can adjust charge carrier concentration and the trapping sites related to defects, facilitating efficient electron transport and improved interaction of biomolecules, thereby enhancing electrode transduction. For example, transition metal dopants incorporated into ZnO could provide active redox centers, improving the electrochemical detection of biomolecules.

**Figure 4:**
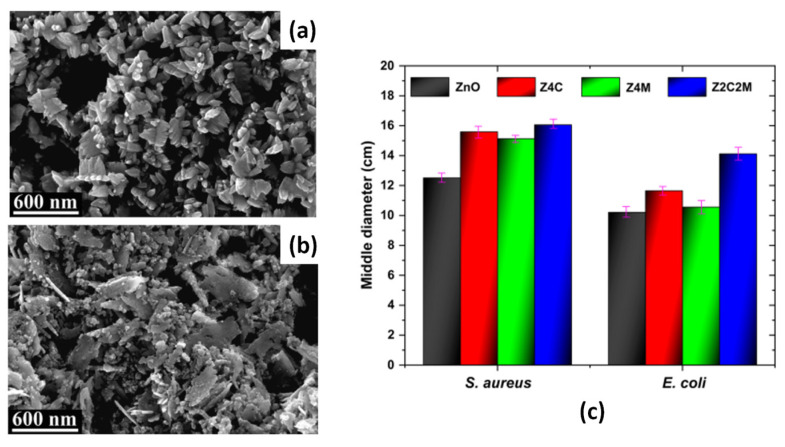
SEM micrographs of pure ZnO (**a**) and Co+Mn-co-doped ZnO (**b**) samples and their inhibition halos against of *E. coli* and *S. aureus* bacteria (**c**). The inhibition halos of Co-doped ZnO (Zn4C) and Mn-doped ZnO (Z4M) are included. Adapted with permission from [130].

In [130], Co^2+^- and Mn^2^-co-doped ZnO nanoparticles were synthesized by the sonochemical method, and their photocatalytic and antimicrobial properties were studied. The SEM micrographs revealed the growth of ZnO particles in a nano-plate morphology (Figure 4a), which transformed into semi-spherical morphology with smaller sized nanoparticles in the case of single-doping with Co or Mn. Dual doping of ZnO resulted in a surface morphology where the semi-spherical nanoparticles coexisted with the nanoplates (Figure 4b). The observed increase in photocatalytic activity of the co-doped ZnO sample, compared to pure or single-doped ones, was attributed to the greater number of oxygen vacancy defects, which prevented the electron–hole recombination process. The antimicrobial activity of the ZnO powders in [130] was examined against *Escherichia coli* (*E. coli*) and *Staphylococcus aureus* (*S. aureus*) bacteria by the diffusion disk technique, and the results are presented in Figure 4c [130]. In Figure 4c, the samples of pure ZnO, Co-doped ZnO, Mn-doped ZnO, and the dual (Co, Mn)-doped ZnO are denoted as ZnO, Zn4C, Z4M, and Z2C2M, respectively. For both bacteria, the antimicrobial capacity of the samples increases with doping, as the highest mean values of the inhibition halos were recorded for the dual-doped samples (Figure 4c) [130].

To develop a highly stable and sensitive DNA biosensor for the detection of meningitis, a nickel-doped ZnO thin-film matrix was employed [131]. The sensor was fabricated by immobilizing a 23-mer oligonucleotide sequence on the Ni-ZnO/ITO electrode through electrostatic interaction. SEM analysis revealed nanostructured surface morphology that facilitated the loading of single-stranded thiolate DNA. Electrochemical studies using methylene blue-mediated DPV demonstrated a wide linear detection range (5–200 ng/μL), high sensitivity (49.95 µA/decade), low detection limit (5 ng/μL), and a fast hybridization time of 30 s. The detection response was further validated by electrochemical impedance spectroscopy (EIS). In this case, oligonucleotides can be considered as co-dopants.

For the qualitative detection of cardiac troponin-T (cTnT) cardiac biomarker, DNA aptamer-functionalized ZnO thin films have been proposed in [132]. In this research, the ZnO surfaces were biofunctionalized using biotin–streptavidin chemistry. The aptamer-cTnT interaction was evaluated by Kelvin force microscopy, which monitored changes in contact potential. To highlight its technological relevance, a ZnO thin-film transistor microdevice was fabricated and successfully used to detect cTnT via aptamer-mediated biofunctionalization [132].

All the scientific studies [117,118,119,120,121,122,123,124,125,126,127,128,129,130,131,132] quoted above highlight the essential importance of choosing appropriate methods for synthesis of ZnO nanoparticles with controlled morphology and excellent characteristics for potential performance in biosensors. Advantages of doping of ZnO with biocompatible elements have been shown, offering better immobilization of biomolecules and higher detection accuracy. Scientists and engineers still face challenges in producing biosensors with high stability and long-term reproducibility that provide consistent, accurate, and precise measurements from different batches of biosensors.

### 3.3. UV Photodetectors

Doping with rare earth co-elements (e.g., Er+Al) improves UV reactivity by reducing the dark current and increasing the photoconductivity [23,133].

**Figure 5:**
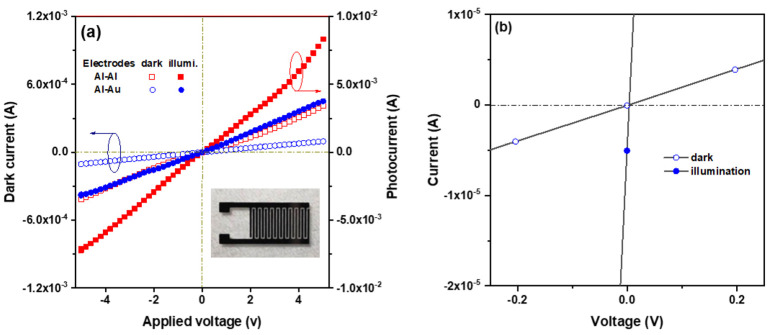
Current–voltage (I-V) characteristics of the ZnO:Ga-In MSM UV photodetectors in the dark and under UV illumination conditions (**a**) with symmetric electrodes (Al and Al) and asymmetric electrodes (Al and Au) and (**b**) with Al-Au asymmetric electrodes in the voltage range from −0.2 V to 0.2 V. The inset in (**a**) shows a digital photograph of the Al-Au devices. Reproduced from [134].

**Figure 6:**
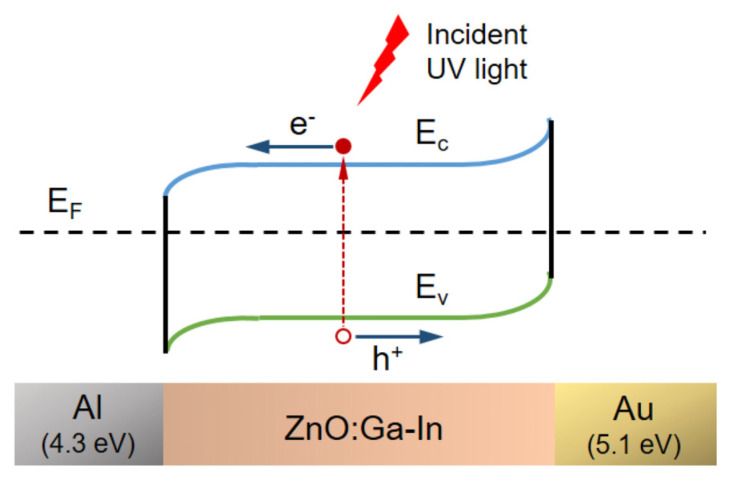
Schematic representation of the energy band diagram for the n-type Ga+In-co-doped ZnO semiconductor with Al–Au asymmetric electrodes, shown both at zero bias and under UV illumination. Reproduced from [134].

Ga- and In-co-doped ZnO thin films were prepared via sol–gel spin coating on Corning glass substrates to fabricate metal–semiconductor–metal (MSM) ultraviolet photodetectors (PDs) with either symmetric Al-Al or asymmetric Al-Au interdigital electrodes [134]. The asymmetric Al/ZnO:Ga-In/Au structure exploits the work function difference between Al and Au to enable self-powered UV detection. Compared to symmetric MSM-PDs, asymmetric devices showed an enhanced photo response at 5 V bias and generated a photocurrent (6.0 × 10^−5^ A) even at zero bias, with a sensitivity of 0.25 and a responsivity of 0.03 mA/W. This demonstrates that asymmetric electrodes are effective for self-powered UV photodetection. Furthermore, Figure 5 shows that the device with asymmetric electrodes produced a low photocurrent under UVA illumination when no bias voltage was applied (solid circle). The energy band diagram, shown in Figure 6, illustrates the use of two metals with different work functions, Al and Au, as asymmetric contacts for the n-type Ga+In-co-doped ZnO semiconductor layer [134].

### 3.4. Solar Energy Harvesting

ZnO is a promising material in thin-film solar cells, acting as a transparent conducting oxide (TCO), a buffer layer, or an electron transport layer. Its high transparency, good conductivity, and favorable band alignment are key advantages.

The effects of co-doping are generally manifested by

- Higher conductivity and transparency: Doping with co-dopants (e.g., Al+In, Ga+Sn) increases the carrier concentration while minimizing optical absorption losses.
- Bandgap tuning: A slight narrowing or broadening of the bandgap by double dopants optimizes sunlight absorption.
- Defect passivation: Reduction in recombination centers, increasing charge collection efficiency.
- Improved surface texture and morphology: Enhanced light capture and reduced reflection.

Through combined in situ AC electrical conductivity and capacitance measurements, it was possible to reveal the presence of mobile species, specifically proton carriers, on the alumina surface at low temperatures [135].

Targeting various solar energy harvesting applications:

(i) As a transparent conducting oxide (TCO), ZnO thin film serves as a cost-effective alternative to indium tin oxide (ITO), as a transparent electrode. Doping improves the stability of zinc oxide films at high temperatures and leads to an enhancement in their conductivity [136,137,138].
(ii) As a buffer/window layer in thin-film solar cells (e.g., CIGS, CdTe), ZnO acts as a window layer, transmitting light while forming a junction with the absorber [139,140,141].
(iii) As an electron transport layer (ETL) in perovskite and dye-sensitized solar cells, ZnO improves electron collection efficiency [142,143].
(iv) In the form of nanostructured ZnO (nanorods, nanowires), it improves light harvesting and increases the surface area for dye loading in dye-sensitized solar cells (DSSCs) [144,145,146,147].
(v) In photoelectrochemical cells (PECs), ZnO thin films are used as photoanodes for solar-driven water splitting [148,149,150].

**Figure 7:**
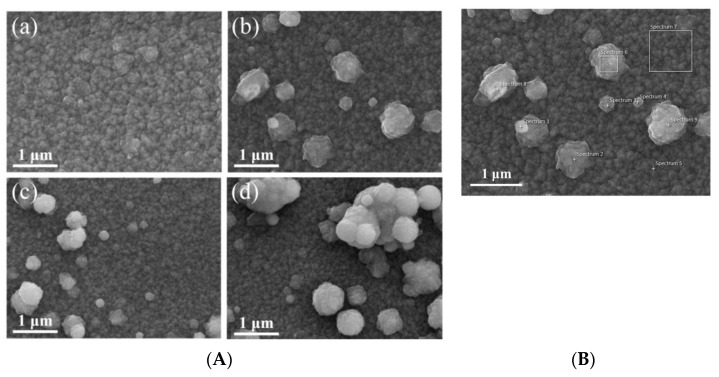
SEM image (**A**) of films with different Zr concentrations: (**a**) GZO (0 at%); (**b**) GZO:Zr (2 at%); (**c**) GZO:Zr (4 at%); (**d**) GZO:Zr (6 at%), and (**B**) GZO:Zr (2 at%). Reproduced from [151].

In [151], co-doping of ZnO was investigated to obtain materials with improved electrical properties, better-controlled optical properties, and with a higher degree of opacity. Ga and Zr were chosen as dopants, and the materials were synthesized using atmospheric pressure plasma jet (APPJ) systems [151]. SEM investigation of the surface morphology highlighted the appearance of spherical particles on the surface of the films, which became bigger with increases in the Zr concentration. These findings demonstrate that ZrO_2_ grown on the surface of a film can significantly increase its opacity. Therefore, by correlating the Zr concentration with the targeted properties, films with the desired opacity and certain optoelectronic properties can be produced. In Figure 7A, SEM images of ZnO films doped with different concentrations of Ga and Zr are presented. Also, in Figure 7B, EDS analysis revealed the chemical composition of the spherical particles on the film surface. This suggests that the presence of these particles increases the surface area of the film and consequently leads to a higher probability of adsorption of airborne contaminants. This method simplifies the process of preparing high-quality hazy films, useful in solar cell applications [151].

**Figure 8:**
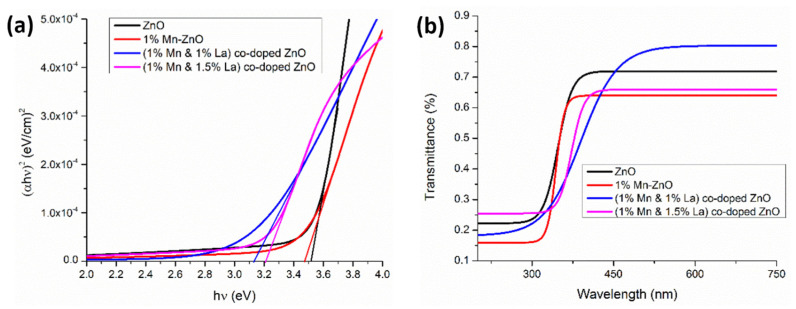
(**a**) Band gap energy and (**b**) transmittance of undoped ZnO films and co-doped ZnO films with 1% Mn and (1% and 1.5%) La. Reproduced from [152].

Amjad et al. [152] prepared thin films of Mn-doped ZnO and ZnO co-doped with 1% Mn and 0.5%, 1%, or 1.5% La using the sol–gel dip coating technique. Numerous structural properties were investigated, and various optical parameters such as band gap, absorption, refractive index, transmittance, and dielectric constants were calculated. Dye-sensitized solar cells (DSSCs) were prepared using these films. The results indicate a decrease in *E_g_* for the films co-doped with 1% La + 1% Mn, but an increase in *E_g_* for those co-doped with 1.5% La + 1% Mn (Figure 8a). The plot of the transmittance spectra (Figure 8b) indicates that the ZnO films co-doped with 1% Mn + 1% La are highly transparent in the visible region, which is a suitable characteristic for DSSCs [152].

**Figure 9:**
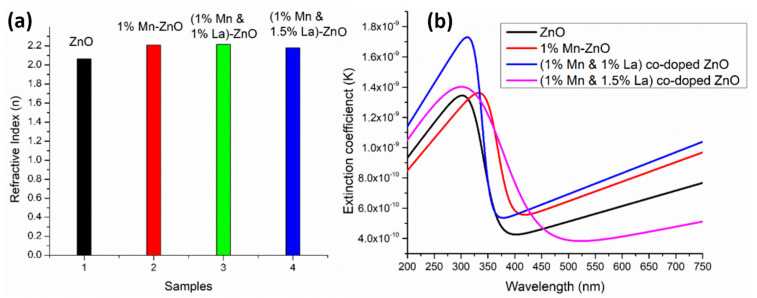
Comparison of (**a**) refractive index and (**b**) extinction coefficient for undoped ZnO thin films and Mn+La-co-doped ZnO thin films. Reproduced from [152].

**Figure 10:**
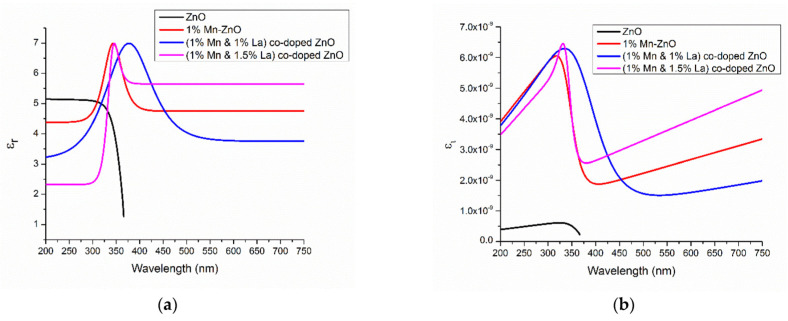
Variation in the dielectric constants of (**a**) εr and (**b**) εi of undoped ZnO thin films and Mn+La-co-doped ZnO thin films. Reproduced from [152].

The average values of the refractive index in Figure 9a and the spectral dependences of the extinction coefficient (Figure 9b) and dielectric constants (Figure 10) indicate that the 1% Mn + 1% La-co-doped ZnO film exhibits the maximal light scattering and is suitable for solar cell applications [152].

In conclusion, for comparison, a DSSC prepared with the ZnO film co-doped with 1% Mn + 1% La has an efficiency of 1.89%, which is 174% higher than that of the undoped ZnO-based DSSC.

### 3.5. Optical Applications

The intrinsic optical properties of ZnO (such as high transparency, strong UV emission, and nonlinear optical response) make it an excellent candidate for next-generation optical devices. Dual doping unlocks additional functionalities. Thus, some of the benefits shown by co-doping ZnO films are mainly the following [153,154]:

(i) Tunable refractive index: Essential for waveguides, antireflection coatings, and photonic crystals.
(ii) Enhanced photoluminescence: Co-doping of the oxide with rare earth elements (e.g., Eu+Al, Er+Ga) produces multicolor emission (visible and infrared) for display and lighting applications.
(iii) Enhanced nonlinear optical effects: Useful in frequency doubling and optical modulation.
(iv) Magneto-optical properties: Co-doping with transition metals (e.g., Co+Mn) confers magneto-optical activity to insulators and modulators.

Optical applications of co-doped ZnO films:

(i) For UV and visible LEDs, co-oxide doping improves radiative recombination rates.
(ii) In the case of photodetectors, co-doping results in highly sensitive detection in the UV, visible, and even IR regions, with reduced noise.
(iii) Optical coatings provide tunable reflection and absorption characteristics for lasers, sensors, and cameras.
(iv) Photonic integrated circuits can be useful as low-loss waveguides and active optical elements.
(v) Room temperature ferromagnetism (RTFM) effect.

**Figure 11:**
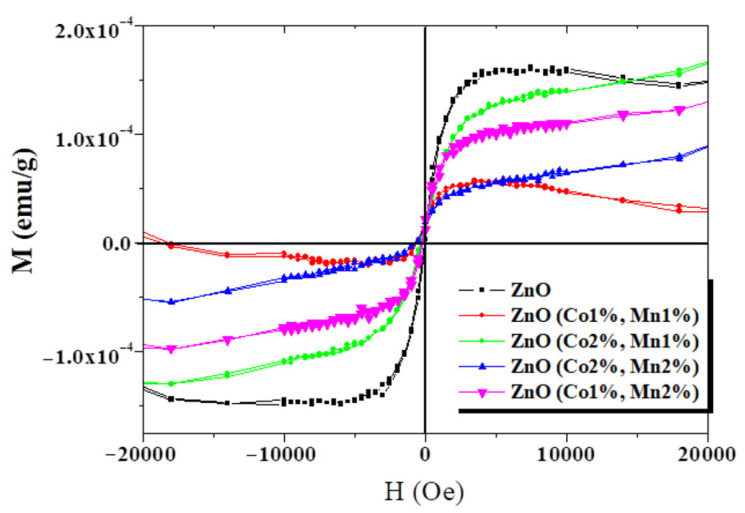
Room-temperature magnetization versus magnetic field for undoped ZnO and Co+Mn-co-doped ZnO samples. Reproduced from [155].

Despite numerous studies on the effect of doping and co-doping on the physical properties of zinc oxide, the origin of room-temperature ferromagnetism (RTFM) remains a highly debated topic. Yahmadi et al. focused on examining the impact of Co and Mn atom incorporation on the physical properties of ZnO films prepared by spray pyrolysis [155]. They provided a detailed characterization of the structural, morphological, optical, and magnetic properties of the co-doped nanocrystalline films. The novelty lies in the fact that both undoped and co-doped (Co+Mn) thin films exhibited ferromagnetism at room temperature (Figure 11), which may be due to the growth by spray pyrolysis [155]. These results suggest potential applications in the field of spintronic devices.

Although Co+Mn-co-doped ZnO thin films exhibit ferromagnetism with an increased magnetic moment, in the case of n-type Co-doped ZnO films, additional charge carriers are insufficient to induce ferromagnetism. To enhance the magnetism in ZnO films, alkali elements could be introduced as co-dopants. The research in [156] focused on the effect of the additional introduction of alkali (Li, Na, and K) elements on the structural, optical, and morphological characteristics of thin films of Co-doped ZnO, deposited using the sol–gel spin-coating technique. It was shown that the layers have a wurtzite structure with a [002] fibrous texture. From the optical studies, it was concluded that alkali atoms replace Co^2+^ in the ZnO lattice. Depending on the inclusion of different alkali elements in the Co-doped ZnO films, different defect emissions in the photoluminescence spectra were registered. Magnetic force microscopy displayed the distribution of the magnetic field and the existence of Co metallic clusters in the grown ZnO thin films.

**Figure 12:**
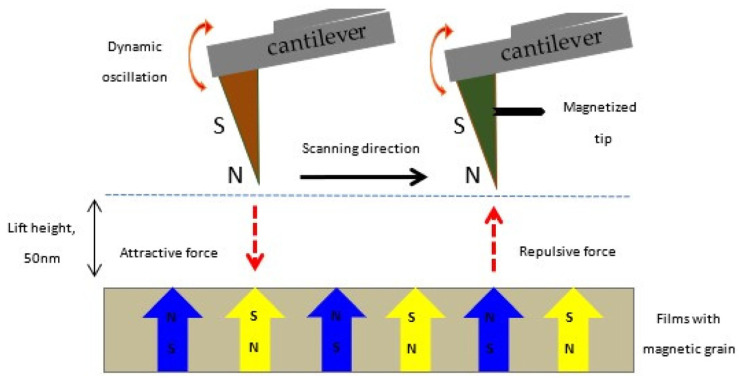
Schematic illustration of the MFM imaging process for ZnO-based DMS films. Magnetic poles, North (N, up) and South (S, down), indicate the magnetization in both the films and the probing tip. Reproduced from [157].

Raship et al. provided a comprehensive analysis of how the concentration of co-dopants, namely Gd and Al, influences the physical and electrical properties of the film, as well as the structures of their magnetic domains [157]. A schematic model of the magnetization orientation of the magnetic pole tips during magnetic force microscopic (MFM) imaging is shown in Figure 12. MFM measurements confirmed the presence of room-temperature ferromagnetism and spin polarization in ZnO films doped with Gd and co-doped with Gd and Al.

**Figure 13:**
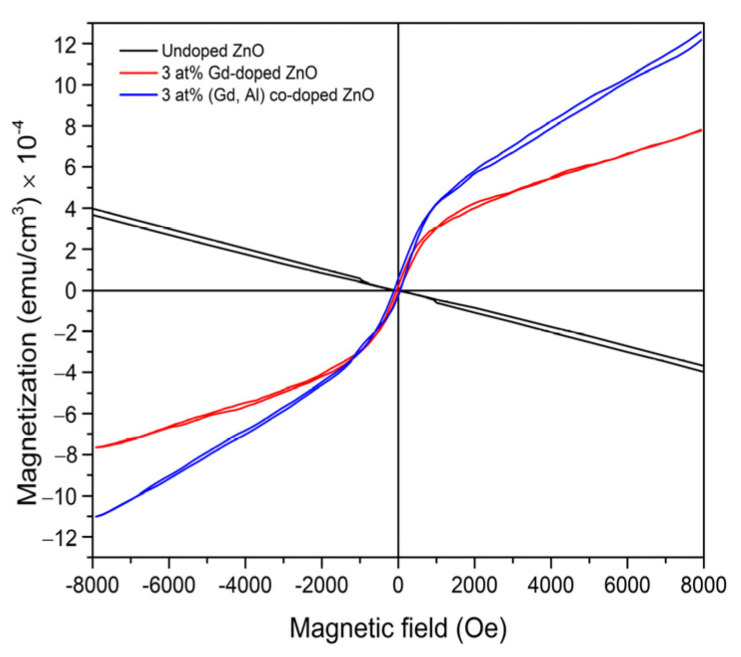
Magnetization versus magnetic field (M–H) curves for undoped ZnO, 3 at% Gd-doped ZnO, and 3 at% (Gd, Al) co-doped ZnO. Reproduced from [157].

Figure 13 shows the M-H curves obtained at room temperature using Vibrating Sample Magnetometer (VSM) equipment, representing the measurement of magnetization as a function of magnetic field in the range from −8 kOe to +8 kOe [157]. Room-temperature M–H measurements further confirm that the incorporation of the Al shallow donor enhances the saturation magnetization in Gd-doped ZnO.

**Figure 14:**
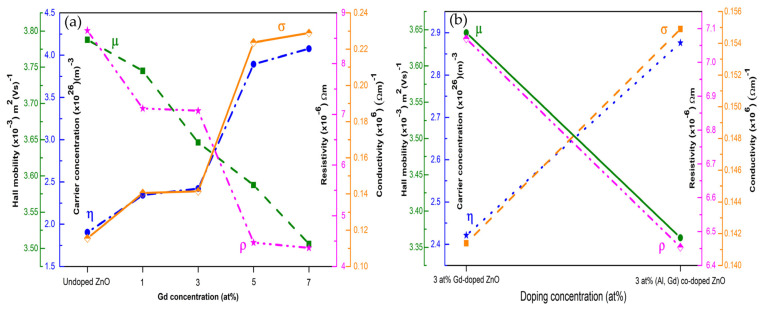
Variation in Hall mobility, carrier concentration, resistivity, and conductivity for (**a**) undoped ZnO and Gd-doped ZnO films, and (**b**) 3 at% Gd-doped ZnO compared to 3 at% (Gd, Al) co-doped ZnO films. Reproduced from [157].

Figure 14 presents the variation in carrier concentration, Hall mobility, resistivity, and conductivity with different Gd concentrations, whereas Figure 14b compares the electrical properties of 3 at% Gd-doped ZnO and 3 at% (Gd, Al) co-doped ZnO films [157].

Wide-bandgap metal oxide semiconductors are promising for optoelectronic, photovoltaic, and transparent electronic applications, including thin-film solar cells, ultraviolet photodetectors, transparent resistive random-access memory (RRAM), short-wavelength light-emitting diodes (LEDs), and field-effect transistors. However, realizing devices such as LEDs, p–n junction photodetectors, and complementary metal-oxide-semiconductor (CMOS) circuits requires both n-type and p-type semiconductors. The scarcity of stable p-type zinc oxide semiconductors remains a major limitation, reducing the efficiency of p–n junction and bipolar devices. It is difficult to prepare p-type ZnO with high concentration of dopants due to the low solubility of the acceptor atoms and their inactivation through formation of donor-like centers to compensate the substitutional acceptors. Because of the nonstoichiometric and n-type behavior of undoped ZnO, strong self-compensation is also observed in p-type ZnO, which originates from intrinsic defects and unintentionally introduced donor-like point defects. All these are serious obstacles for producing reliable, reproducible, and stable p-type ZnO-based devices. Thus, developing stable and reproducible p-type semiconductor thin films is essential.

**Figure 15:**
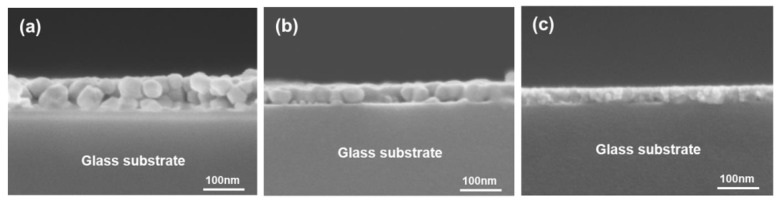
Cross-sectional FESEM images of ZnO-based thin films: (**a**) undoped, (**b**) N-doped, and (**c**) Ga-N-co-doped samples. Reproduced from [158].

To address these limitations, ZnO was doped with various elements, such as Li, Na, N, P, and As, which were tested as acceptor dopants. Co-doping ZnO with elements, such as In, Al, and Ga, proved to be an excellent approach to improve the functional properties of zinc oxide. In [63], a comprehensive study on the electronic structure of defects of Al-doped (n-type) and Al+N-co-doped (p-type) ZnO films and nanorods, synthesized by different methods and theoretical calculations, is presented. Transparent p-type ZnO thin films were fabricated on glass substrates using the sol–gel spin-coating method by N doping and Ga and N co-doping [158]. The prepared materials were comparatively investigated in terms of microstructural and electrical characteristics, as well as optical properties of undoped, N-doped, and Ga+N co-doped thin films. The incorporation of N and Ga+N into ZnO thin films resulted in finer microstructures with lower surface roughness (Figure 15) [158].

**Figure 16:**
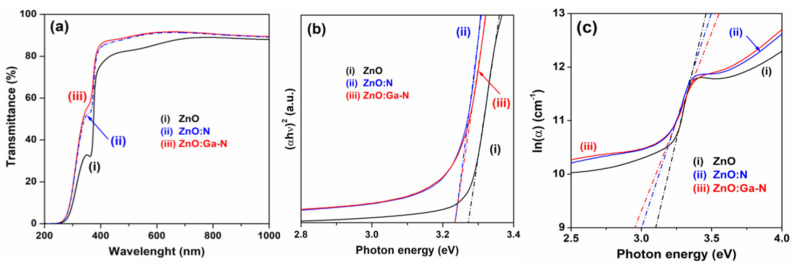
(**a**) Optical transmission spectra, (**b**) Tauc plot of (αhν)^2^ versus photon energy (hν), and (**c**) plot of ln(α) versus photon energy (hν) for ZnO, ZnO:N, and Ga+N co-doped ZnO thin film samples. Reproduced from [158].

Similarly, the recorded optical transmittance spectra indicated an improved optical transparency in the visible range (Figure 16) [158].

**Figure 17:**
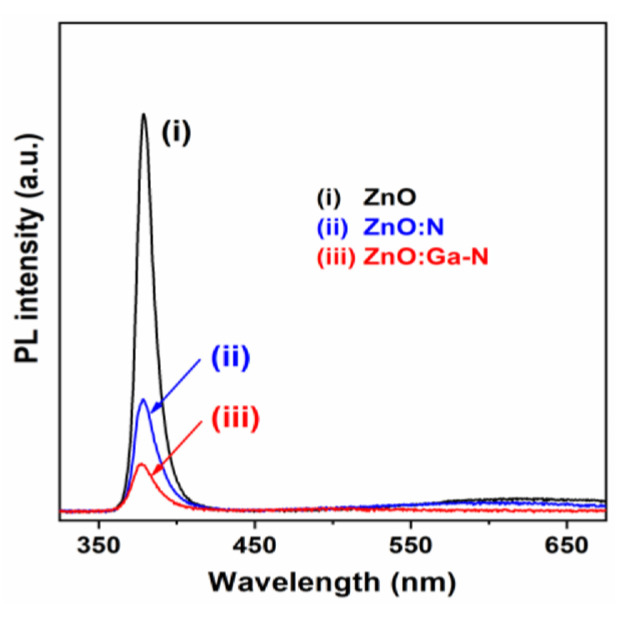
Room-temperature photoluminescence (PL) spectra comparison of undoped ZnO, N-doped ZnO:N, and Ga+N-co-doped ZnO thin film samples. Reproduced from [158].

XPS analysis confirmed the successful incorporation of N and Ga+N into ZnO thin films [158]. PL spectra at room temperature revealed near-band edge and deep-level emissions (Figure 17). Hall measurements showed that N-doping converted ZnO from n-type to p-type, yielding a hole concentration of 1.83 × 10^15^ cm^−3^ and a resistivity of 385.4 Ω·cm. In contrast, Ga and N0co-doped films exhibited stronger p-type conductivity, with a higher hole concentration (~4.0 × 10^17^ cm^−3^), lower resistivity (5.09 Ω·cm), and more stable conduction (for over 3 weeks) compared to N-doped films.

**Figure 18:**
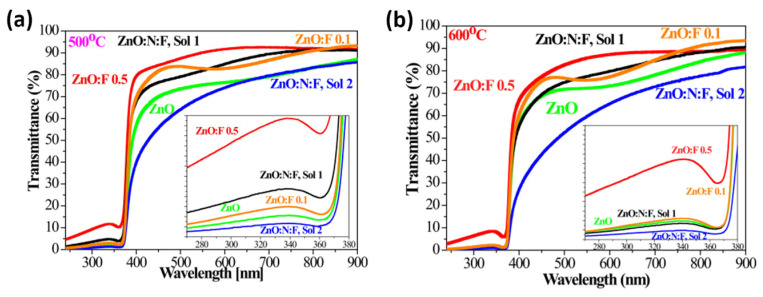
Optical transmittance in the range of 280–900 nm of undoped, doped, and co-doped ZnO films annealed at (**a**) 500 °C and (**b**) 600 °C. Insets highlight the excitonic features of the films. Reproduced from [159].

Ivanova et al. reported the preparation of N and F-co-doped ZnO thin films using the sol–gel spin-coating method, deposited on Si and quartz substrates [159]. This study analyzed the impact of two different dopants, F and N doping (both individual and dual), and thermal treatments on the structural, vibrational, and optical behavior of ZnO films. N+F-co-doped ZnO thin films were prepared using two precursor solutions, denoted as sol 1 (0.1% F) and sol 2 (0.5% F). An improvement in transparency was observed for co-doped ZnO films compared to undoped ZnO, reaching a transmittance of over 82% and a reflectance of less than 8.5% in the visible spectrum. The transmittance spectra presented in Figure 18 demonstrate that both doping and co-doping significantly influence the optical response of ZnO films [159]. The pronounced excitonic absorption observed in the doped samples is indicative of their high structural quality, as the excitonic features are strongly correlated with the crystalline order and integrity of the film.

**Figure 19:**
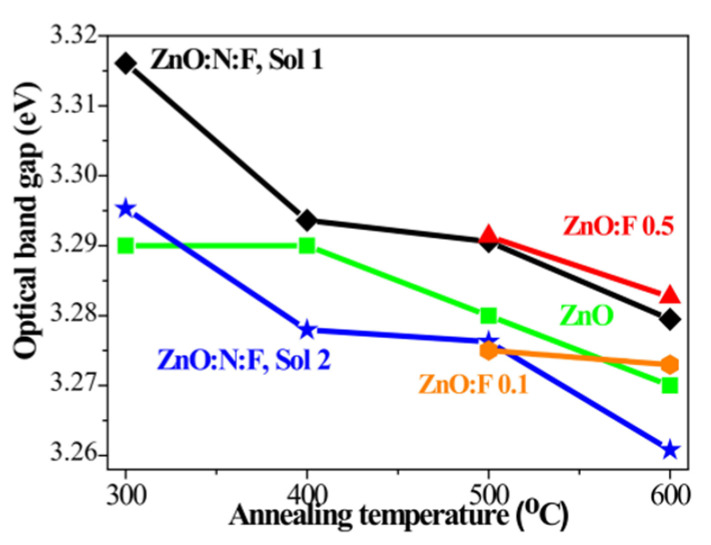
Variation in the optical band gap in undoped ZnO, F0doped ZnO, and N+F-co-doped ZnO thin films subjected to different annealing temperatures. Reproduced from [159].

The effect of annealing temperature on the optical band gap values of undoped ZnO, F-doped ZnO, and N+F-co-doped ZnO films is shown in Figure 19. It is observed that the band gap of the co-doped samples is wider than that of the other films [159].

**Figure 20:**
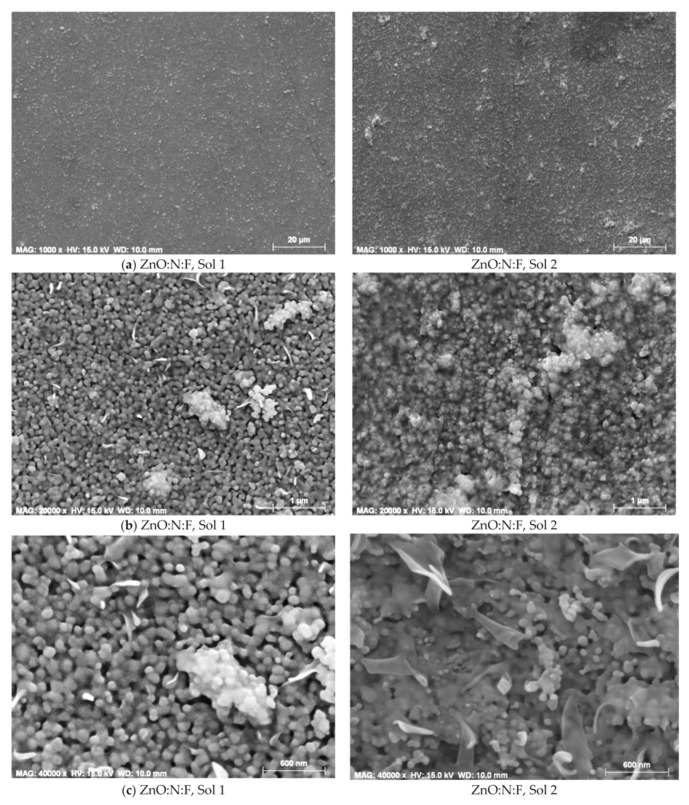
FESEM images of N+F co-doped ZnO films synthesized from sol 1 and sol 2 and annealed at 600 °C, at magnifications of (**a**) 1000×, (**b**) 20,000× and (**c**) 40,000×. Reproduced from [159].

FESEM analysis showed that co-doping with N and F resulted in surface morphologies characterized by spherical and irregular grains, along with a porous texture. The introduction of co-dopants also altered the wrinkle-like features typically observed in ZnO films, in both the N+F co-doped ZnO thin films, prepared with solution 1 (0.1 wt% F) and solution 2 (0.5 wt% F), annealed at 600 °C (Figure 20) [159]. N- and F-co-doped ZnO films synthesized via the sol–gel method thus show promise for optoelectronic device applications.

**Figure 21:**
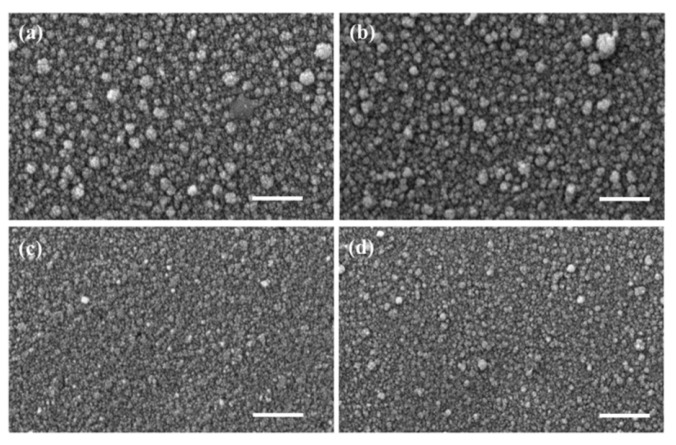
SEM images of ZnO thin films: (**a**) undoped, (**b**) doped with 5% Al, (**c**) doped with 5% Co, and (**d**) co-doped with 5% Al and Co. Scale bar: 1 μm. Reproduced from [160].

A new versatile electrospray deposition method was employed to fabricate ZnO thin films doped with cobalt (CZO) (5%) and co-doped with Co (2.5%) and Al (2.5%) (CAZO) [160]. This study performed a comparative analysis of the doped and co-doped thin films, examining their structural, morphological, and surface characteristics, as well as optical properties, including refractive index, absorption coefficient, optical band gap, and Urbach energy. Uniform polycrystalline thin films with an approximate thickness of 200 nm and free of cracks were successfully fabricated. Cobalt doping resulted in a significant reduction in grain size for both undoped and co-doped ZnO, while simultaneously increasing the crystallite size along the (101) orientation (Figure 21) [160].

**Figure 22:**
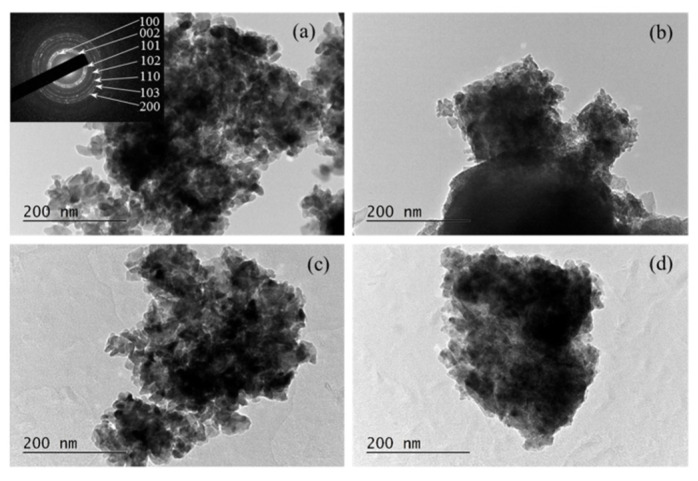
TEM images of ZnO thin films: (**a**) undoped, (**b**) 5% Al-doped (AZO), (**c**) 5% Co-doped (CZO), and (**d**) co-doped with 5% Al+Co (CAZO). The inset shows the corresponding SAED pattern with indexed diffractions. Reproduced from [160].

TEM analysis, complemented by selected area electron diffraction (SAED), revealed that all films exhibited a similar morphology, composed of irregularly shaped nano-crystallites, with sizes between 20 and 30 nm (Figure 22) [160]. Both techniques further confirmed that 5% doping induces lattice contraction resulting from the substitution of Zn ions with the smaller Al and Co ions.

The as-deposited doped ZnO films obtained through the electrospray method could serve as functional materials and present potential applicability in optoelectronics, spintronics, and photocatalysis.

TCOs are vital for optoelectronic devices like LEDs, displays, and solar cells due to their high conductivity and transparency. Indium tin oxide (ITO) is widely used but limited by indium’s toxicity, scarcity, and cost. To overcome these issues, ZnO-based TCOs doped with elements such as In and Ga are explored, as they can substitute Zn^2+^, create oxygen vacancies, and markedly enhance conductivity, even at low concentrations [161]. Co-doping improves both electrical performance and chemical stability. Additionally, optical annealing techniques (e.g., rapid thermal, direct irradiation, or laser processing) offer an effective alternative to conventional annealing, enhancing conductivity, stress relief, and surface properties of ZnO thin films.

**Figure 23:**
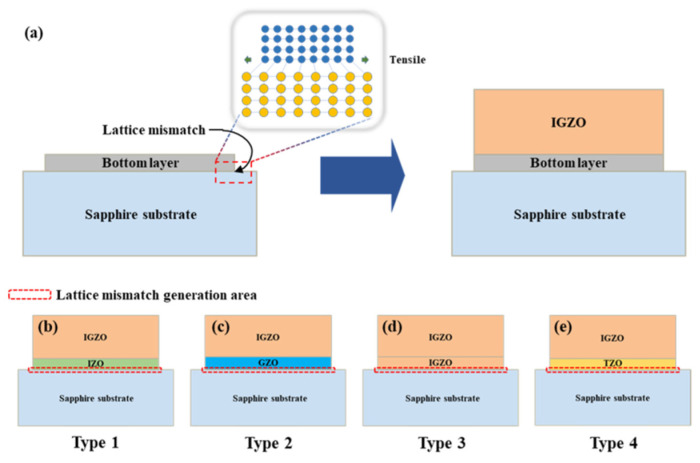
The diagram of the thin film structure (**a**) and schematic presentation of four (In+Ga) co-doped ZnO multilayer thin-film types: Type 1 (**b**), Type 2 (**c**), Type 3 (**d**), and Type 4 (**e**). Reproduced from [162].

Yun et al. used In and Ga as dopant ions for patterning lattice mismatch modeling [162]. Four different types of (In+Ga) co-doped ZnO multi-layer thin films were fabricated on sapphire substrates using solution synthesis, spin coating, and CO_2_ laser annealing (Figure 23). Films with a Ga-doped ZnO bottom layer exhibited the lowest lattice mismatch with the substrate. This study used solution synthesis and spin coating to deposit In+Ga-co-doped thin films on sapphire, offering low cost, large area coverage, and precise control of composition and thickness. In addition, the effects of CO_2_ laser annealing on thin films with varying lattice mismatches, depending on the type of bottom layer to the substrate, were examined [162]. The results showed that optimizing the mismatch can improve the annealing results of thin films for applications in optical devices.

### 3.6. Summary

This review has summarized an overview of the co-doping of ZnO thin films. The limitations of pure ZnO for its widespread use in advanced sensing technologies have been presented. The method of co-doping ZnO thin films with two different elements has been highlighted, which has emerged as an effective strategy to improve the structural, electronic, and surface properties of ZnO. In addition, this review summarized recent advances in co-doped ZnO thin films, highlighting how various combinations of dopants improve carrier concentration and adsorption behavior. Synthesis methods, dopant incorporation mechanisms, and performance correlations, as well as challenges such as dopant clustering and scalability, were also discussed.

The main problem with doping ZnO thin films with one or two elements is that concentrations above 7–8 percent can lead to deterioration of the structural and electrical properties. This is because the dopant atoms can cause some distortions in the electrostatic lattice, form insulating precipitates (such as Al_2_O_3_), or lead to the formation of compensating defects, reducing the efficiency of doping [35,154]:

(i) When the dopant concentration is too high, the crystal structure can be negatively affected. For example, adding too much indium (In^3+^) can cause problems, because its ionic radius is much larger than that of zinc (Zn^2+^), which makes it difficult to incorporate In into the ZnO lattice, and the limited oxygen tetrahedral environment of the wurtzite structure cannot accommodate a high concentration of these foreign ions.
(ii) Intrinsic defects, such as oxygen vacancies, can act as compensating acceptors, which prevents the desired effect of doping. This effect is particularly pronounced when trying to create p-type ZnO.
(iii) Above a certain concentration, the dopant can aggregate and form its own insulating phases. For example, aluminum can form insulating precipitates (such as Al_2_O_3_) which increases the resistivity and reduces the concentration of charge carriers.
(iv) The combination of the aforementioned effects can lead to a degradation of the desired properties, such as a reduced concentration of charge carriers or an increased resistivity, even if the initial doping is aimed at increasing conductivity.

Because of these challenges, researchers aim to find an “optimal” dopant concentration (e.g., around 4–5 at.% for aluminum, or around 1–3% for indium) at which the films have the best balance between properties, such as high charge carrier concentration and low resistivity, along with good optical transmission.

The key distinction between co-doping and single-doping in ZnO thin films lies in the synergistic effect that arises from the simultaneous incorporation of two different dopant elements. In single-doped ZnO, one type of dopant typically modifies either the electrical or structural characteristics, such as increasing carrier concentration or creating oxygen vacancies, but often at the expense of other properties, like stability or crystallinity. In contrast, co-doping enables complementary interactions between two dopants that collectively optimize multiple parameters. For instance, one dopant can enhance carrier mobility or conductivity by introducing shallow donor levels, while the other can increase the density of active surface sites or control oxygen vacancy formation. This dual modification results in improved charge transport, enhanced adsorption and desorption dynamics of target gas molecules, and greater stability under varying environmental conditions. Moreover, the co-doping approach can mitigate problems commonly encountered in single-doped systems, such as dopant segregation, lattice distortion, or compensation effects, by balancing the charge and size mismatch between dopant ions. As a result, co-doped ZnO thin films exhibit enhanced sensitivity, selectivity, faster response/recovery times, and lower operating temperatures compared to their single-doped counterparts.

Thus, the synergistic effect in co-doping arises from the collaborative tuning of ZnO’s structural, electronic, and surface properties, achieving a level of performance and tunability unattainable through single-doping alone.

It is important to emphasize a few more factors that contribute to the benefits of co-doping.

As demonstrated in reference [163], co-doping offers clear advantages in the case of ZnO. The Hall effect measurements have shown that the introduction of N into ZnO films is not sufficient to compensate the native donor-like impurities in pure ZnO and change the conductivity type from n-type to p-type, but the introduction of 1% In as a co-dopant succeeds in achieving this. The band gap energy also changes significantly from 3.37 eV (pure ZnO) to 3.42–3.57 eV (N+In co-doped ZnO) depending on the annealing temperature [163]. A. Malik and D. Basak have shown how the Hall parameters are improved by co-doping ZnO films with 13 different pairs of cation–cation dopants [164]. It is stated, we quote, that “A resistivity value as low as 10^−4^ Ω·cm (for 2 at% B + 6 at% F), corresponding to the highest carrier concentration with a high transmission value in the visible solar spectrum region, indicates that this co-doping pair is very promising for achieving high conductivity in ZnO films compared with commercial TCOs.”

More examples are presented in Table 1, which compiles data published over the past 10 years. We have presented only one or two examples/year where the pair of dopants, their concentration, and deposition methods are different, depending on the application requirements.

## 4. Challenges and Future Directions

Co-doped ZnO thin films incorporating two distinct dopant elements face several critical challenges. One major issue is achieving a uniform distribution of both dopants at the atomic scale, which is essential for achieving consistent material properties. In addition, the formation of secondary phases or the degradation of optical transparency can occur, complicating the synthesis process. Another key challenge lies in balancing competing material properties, such as maintaining high electrical conductivity while minimizing optical absorption.

Future research directions in this field include the exploration of unconventional dopant combinations to achieve multifunctional behavior. Nano-structuring approaches, such as the fabrication of nanorods or quantum dots, offer promising routes for further performance improvements. The integration of co-doped ZnO films into flexible and transparent substrates is also a promising avenue for applications in wearable electronics. Furthermore, hybrid device architectures combining co-doped ZnO with 2D materials or perovskites could open up new possibilities in optoelectronics. Fundamental research focusing on quantum emission phenomena and spintronic behavior in co-doped systems is expected to deepen the understanding of their underlying physics. Finally, real-world testing to evaluate long-term reliability and durability will be crucial for the practical deployment of these materials.

## 5. Conclusions

This review has demonstrated, with supporting evidence from the selected references, that co-doping ZnO thin films with two different elements is a highly effective strategy for designing multifunctional materials for next-generation sensing, solar energy devices, and optical technologies. The synergistic effects of dual dopants enable unprecedented control over electrical, optical, and surface properties, surpassing the limitations of single-doping approaches.

The challenges faced by the researchers and the future directions for developing advanced ZnO-based materials have been outlined. By optimizing dopant selection, fabrication, and processing, co-doped ZnO thin films can serve as high-performance materials for gas and biosensors, transparent electrodes in photovoltaics, photodetectors, light emitters, and integrated photonic devices. Continued research is expected to unlock even more advanced functionalities, paving the way toward smarter, more efficient, and more sustainable electronic and photonic systems.

## Figures and Tables

**Table 1:** Optical bandgap and Hall parameters of co-doped ZnO films in the selected papers published in the last ten years.

Year	Dopants	Concentration (at.%)	Deposition Method	Eg (eV)	Special Observation	Application	Ref
2017	Ga, In (Ga 3%)	In 0.5% In 1%	Two-step chemical route: sol–gel + hydrothermal	3.28	Influence of annealing 100 °C, 300 °C, and 500 °C on optical and electrical properties: Hall Effect measurements have established that all samples, regardless of annealing temperature, showed p-type conductivity with a carrier concentration of 3.25 × 10^14^ cm^−3^ and a mobility of 2.04 cm^2^V^−1^s^−1^.	Optoelectronic devices: panel displays, sensors for ozone and UV light detection, and flexible electronics applications	[165]
2017	Cr, In	Cr 1%: In 1% Cr 2%: In 1% Cr 1%; In 2%	Spray pyrolysis	3.13 3.17 3.10	High photocatalytic efficiency.	Photocatalytic applications	[166]
2018	Al, Sn (Al 2%)	Sn 1% Sn 2% Sn 3% Sn 5%	Spin coating	3.95 3.68 3.66 3.94	The lowest resistivity value of about 1.65 × 10^−3^ Ω^.^cm was obtained for Al 2 at.% and Sn 2 at.%.	Fabrication of thin-film transistors (TFTs) and ultraviolet light-emitting diodes (UV-LEDs).	[167]
2018	Gd, Al (Al 3%)	Gd 0.5% Gd 1% Gd 1.5%	Nebulizer spray method	3.31 3.30 3.28	Resistivity 3.42 × 10^−4^ Ω·cm for 1.5% Gd.	Optoelectronic applications	[168]
2019	Ce, Al (Al 1%)	Ce 0% Ce 3% Ce 5% Ce 7%	Sol–gel	3.22 3.13 3.20 3.10	The I–V characteristics of the Schottky diodes manifest good rectification behavior at highest doping of Ce (7 at.%) with an ideality factor of 2.40, barrier height of 0.77 eV, and series resistance of 262 Ω.	Schottky diode devices	[169]
2020	F, Ga	1% F + 1% Ga	RF magnetron sputtering	3.49	Resistivity of 6.81 × 10 ^−4^ Ω^.^cm. Carrier concentration of 2.61 × 10^20^ cm^−3^. Mobility of 35.1 cm^2^/ V^−1^s^−1^.	Perovskite solar cell applications	[170]
2021	Co, Ni (Co 0.04%)	Ni 0.03% Ni 0.06% Ni 0.09%	Hydrothermal	*E_g_* decreased (3.37–3.16) eV for all co-doped samples.	Optical parameters, including the optical absorption coefficient (*α*), transmittance *(T)*, skin depth, optical density *(OD)*, extinction coefficient (*k*), refractive index (*n*), optical conductivity (*σ_opt_*), and dielectric constants (*εr, εi*) of the grown thin films were discussed.	Optical applications	[171]
2022	Sn, Ni	1% Sn + 1% Ni	Spin coating	3.26	High electrical conductivity.	TCO	[172]
2023	Al, F	Al 2.5–10% F 0.1–10%	Atomic layer deposition	3.32–3.75	Lowest resistivity up to 1–10 × 10^−3^ Ω·cm. Carrier concentration of 2–3.37 × 10^20^ cm^−3^. Mobility of 10^−14^ cm^2^/V^−1^s^−1^.	Fabrication of high-quality AZO films	[173]
2024	Cu, La	2% Cu + 2% La	Drop casting	3.25–3.28	The gas response for 250 ppm NH_3_ was remarkably enhanced to 341%, and a quicker response/recovery time of 80/10 s was obtained.	ZnO gas sensors Ammonia sensing studies	[174]
2025	Al, Cu	2.4% Al + 6.2% Cu	Ultrasonic spray pyrolysis	3.26	Co-doped films showed enhanced photocatalytic activity, which was related to an enhanced crystalline structure and the type of dopants.	Improved photocatalytic performance	[175]

## Data Availability

The data presented in this study are available on request from the corresponding author.
